# Are Tubular Injury Markers NGAL and KIM-1 Useful in Pediatric Neurogenic Bladder?

**DOI:** 10.3390/jcm10112353

**Published:** 2021-05-27

**Authors:** Joanna Bagińska, Agata Korzeniecka-Kozerska

**Affiliations:** Department of Pediatrics and Nephrology, Medical University of Białystok, 17 Waszyngtona Str, 15-274 Białystok, Poland; iklinped@umb.edu.pl

**Keywords:** neurogenic bladder, myelomeningocele, markers

## Abstract

The lack of early biomarkers of renal damage in children with neurogenic bladder (NB) prompts us to investigate the role of promising proteins: neutrophil gelatinase-associated lipocalin (NGAL) and kidney injury molecule-1 (KIM-1). This prospective analysis was conducted on 58 children with NB and 25 healthy children. We assessed urinary levels of NGAL and KIM-1 in both groups. Age, sex, anthropometric measurements, activity assessment, renal function, and urodynamics parameters were analyzed. The differences between the median uNGAL and uKIM-1 in the NB group compared to control were recorded. However, only uNGAL levels were statistically significantly higher. Statistically significant correlation was found between gender, recurrent urinary tract infections, bladder trabeculation, its compliance, activity assessment, and uNGAL. To conclude, elevated levels of uNGAL may be considered a biomarker of tubular injury in children with NB due to MMC in contrast to uKIM-1.

## 1. Introduction

Neurogenic bladder (NB) due to myelomeningocele (MMC), with an estimated prevalence of 1/700 live births, is a condition strongly associated with multiple disturbances which, untreated, can result in progressive renal damage. This process may occur silently, and eventually leads to chronic renal failure [1]. Hence, NB patients require close monitoring to evaluate the evolution of glomerular and tubular renal function in order to rapidly diagnose and treat the worsening of renal outcome. Identifying the biomarkers that are capable of early detection of renal damage would represent a tremendous advance in the care of NB children. Recently, several biomarkers related to inflammation and tubular injury have been identified as potent predictors of renal outcome, including neutrophil gelatinase-associated lipocalin (NGAL) and kidney injury molecule-1 (KIM-1).

NGAL belongs to the lipocalin proteins, is expressed at very low levels in human tissues, including kidney, lungs or stomach, and its expression increases in inflammation and injured epithelia [2,3]. KIM-1 is a transmembrane glycoprotein highly expressed in regenerating proximal tubular cells [4,5]. Due to their small molecular size, NGAL and KIM-1 are freely filtered and can be easily detected in urine. Urinary NGAL (uNGAL) and urinary KIM-1 (uKIM-1) are rapidly released in response to tubular damage. Therefore, they are very sensitive biomarkers of acute kidney injury (AKI) [4,5,6]. However, it is now increasingly recognized that the inflammatory process and tubular injury have an important role in the pathogenesis and progression of chronic processes, and the severity of tubulointerstitial lesions has a significant impact on renal outcome in, e.g., diabetic nephropathy [7] or chronic kidney disease (CKD) [8,9,10,11,12]. Little is known about tubular injury with regards to pediatric NB. To the best of our knowledge, our study represents the first investigation of uNGAL and uKIM-1 in NB as potential markers in detecting tubular damage.

## 2. Materials and Methods

### 2.1. Patients

This prospective analysis was conducted on 83 children divided into two groups. The study group included 58 children with congenital NB after MMC. We assessed all participants’ medical charts to determine age, sex, anthropometric measurements, and standard deviation scores (WHO z-scores). The presence of urinary tract infection was excluded on the basis of urinary testing and urine culture. A negative C-reactive protein (CRP) excluded current infection. Nonetheless, the history of recurrent urinary tract infections (rUTIs) was recorded. We defined rUTIs as two or more episodes of pyelonephritis, or one episode of pyelonephritis plus one or more episodes of cystitis, or three or more episodes of cystitis, in accordance with the Polish Society of Pediatric Nephrology Guidelines published in 2015 concerning the management of UTI in children.

Renal function parameters included: urinary and serum creatinine (Cr), serum cystatin C (Cys), and glomerular filtration rate (eGFR). Two pediatric equations espoused by the National Kidney Foundation were used to calculate eGFR, incorporating Cr-only (based on the new Schwartz formula: eGFR = 41.3 × (height/Cr)) and Cys-only (eGFR = 70.69 × Cys ^−0.931^). The assessment of Cys was not performed in four children younger than two years of age due to the fact that it is not validated for this age group. The inclusion criterion for the study group was eGFR > 90 mL/min/1.73 m^2^ (in both above-mentioned formulas) to choose NB patients without the biochemical signs of glomerular impairment.

The presence of abnormalities in the upper urinary tract system was assessed by abdominal ultrasound and voiding cystourethrogram. The following ultrasound parameters were evaluated: length and width of the kidneys, bladder wall thickness, hydronephrosis, parenchymal echogenicity, corticomedullary differentiation, and the presence of trabeculation. In the voiding cystourethrogram, we analyzed the presence of vesicoureteral reflux, its grade, laterality, and activity. We divided NB children into three groups: 1—Without VUR, 2—With present VUR, and 3—With a history of VUR in the past.

The assessment of the lower urinary tract system included urodynamic study where the measurements of bladder wall compliance (comp), detrusor pressure at urgency (Pdet urg), cystometric capacity (CC), and maximum detrusor pressure on voiding phase (max p det) were recorded. Patients with high-pressure bladders were included in the study after the appropriate treatment with anticholinergics. The urine samples were collected from them when the bladder pressures had decreased. In this way, we aimed to avoid the influence of high bladder pressures on biomarkers concentrations.

The ambulatory function of MMC patients was defined according to Hoffer’s scale (HS) using the four categories of community: 1HS—Nonambulator, 2HS—Nonfunctional ambulator, 3HS—Household walkers, and 4HS—Community walkers [13]. The lesion level in MMC patients was reported intraoperatively and radiologically and scored from 1 to 3 (1—Thoracolumbar, 2—Lumbosacral, 3—Sacral lesion).

The control group consisted of 25 healthy children who visited a pediatrician for balance tests and had no abnormalities in the urinary or nervous systems.

### 2.2. Biochemistry

First, morning void spot urine samples were collected for measurement of KIM-1 and NGAL. The levels of biomarkers were measured for KIM 1 using BioAssay Human KIM-1 ELISA) and for NGAL using BioVendor Human Lipocalin-2/NGAL ELISA, according to the manufacturer’s instructions. The urinary Cr concentration was used to normalize NGAL and KIM-1 measurements to account for the influence of urinary dilution on concentration. Urinary levels of the biomarkers were expressed as uNGAL/Cr and uKIM-1/Cr ratios (ng/mg Cr).

### 2.3. Statistics

The data were collected in a Microsoft Excel database. Statistical analysis was performed using Statistica 13.0 (StatSoft Inc, Tulsa, OK, USA). Continuous variables were expressed as the median and range, unless stated otherwise. All studied parameters were analyzed using nonparametric tests: Mann–Whitney, Kruskal–Wallis and Chi2 analysis. Correlations were assessed with the Spearman test. Values of *p* < 0.05 were considered significant.

### 2.4. Ethical Issues

This study was approved by the Ethics Committee of the Medical University of Bialystok (R-I-002/105/2018) which complies with the World Medical Association Declaration of Helsinki regarding ethical conduct of research involving human subjects and/or animals. Patients and their caregivers were enrolled in the study after obtaining informed consent.

## 3. Results

The characteristics of the studied children are presented in Table 1. The median age of the enrolled patients was 10 (0.58–17.7) years. There were no differences in age, sex, weight-to-age z-scores, and BMI-to-age z-scores, excluding height-to-age z-scores. This resulted from shorter vertebral dimensions and malformations of the bone structure due to MMC in NB group. We found statistically significant differences in the urinary and serum Cr concentrations and eGFR Schwartz between the studied groups.

Additionally, in the NB children we assessed the serum Cys, with a median concentration of 0.63 (0.18–0.85) mg/L. The eGFR formula with the use of Cys was equal to 105 (90–162) mL/min/1.73 m^2^.

A comparison of the biochemical indices is presented in Table 2. As shown, children with NB have significantly elevated urinary NGAL levels in contrast to healthy participants. We recorded differences in the levels of urinary KIM-1 between the studied groups, but they were not statistically significant.

There were no statistically significant differences between girls and boys participating in the study, excluding the uNGAL/Cr ratio (*p* = 0.02; *p* < 0.05). In both studied groups, a higher uNGAL/Cr ratio was observed in girls. In NB girls, a median of 45 (3.0–240) ng/mg in the uNGAL/Cr ratio was recorded in contrast to 18 (1.07–213.39) ng/mg in the NB boys. In the control group, a median uNGAL/Cr ratio in girls equaled 1.63 (0–93) ng/mL in comparison to 0 (0–3.95) ng/mL in boys.

### 3.1. NB Children

#### 3.1.1. History of Urinary Tract Infections

The majority of NB patients (38/58 (65%)) had no history of rUTIs in contrast to 20/62 (35%) with rUTIs in the past. A comparison of the studied parameters revealed statistically significant differences between NB children with and without rUTIs (*p* = 0.014; *p* < 0.05). The active UTI was an exclusion criterion. However, children who had rUTIs tended to have a higher uNGAL/Cr ratio with a median of 45.13 (7.06–240.1) ng/mg in contrast to children without rUTI history with a median of 19.05 (1.08–213.4) ng/mg.

#### 3.1.2. Assessment of Urinary Tract

Children without VUR constituted 72% (42/58) of the NB group. VUR was present in 9/58 (15%) children, and 7/58 (13%) patients had a history of VUR in the past. The comparison of KIM-1 and NGAL in the above-mentioned groups did not reveal statistically significant differences (Chi2 = 3, *p* = 0.214; Chi2 = 2.22, *p* = 0.327, respectively).

**Figure 1:**
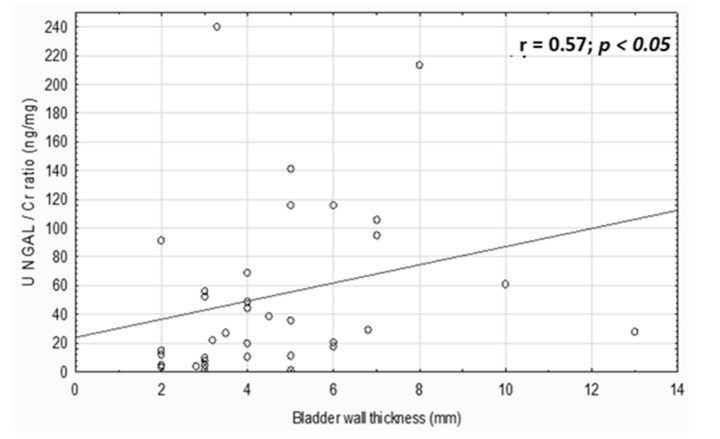
Correlation between uNGAL/Cr ratio and bladder wall thickness.

The increase in uNGAL concentrations was associated with bladder trabeculation (*p* = 0.014; *p* < 0.05). In children with trabeculated bladder, the median of NGAL concentration was 32.13 (3.43–73) ng/mL in contrast to children without the trabeculation with a median of 4.93 (0.55–5.11) ng/mL. Additionally, the uNGAL/Cr ratio positively correlated with the bladder wall thickness (r = 0.57, *p* < 0.05, Figure 1).

**Figure 2:**
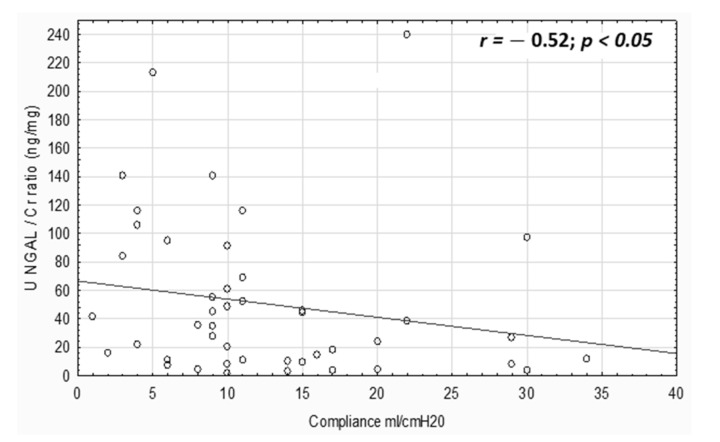
Correlation between uNGAL/Cr ratio and bladder compliance.

In urodynamics, we evaluated Pdet urg (median 20 (5–60) cm H_2_O), CC (median 155 (20–300) mL), comp (median 9 (1–40) mL/cm H_2_O) and max p det (median 12 (1–58) cm H_2_O). No correlation was observed between bladder pressures and the investigated biomarkers. However, the uNGAL/Cr ratio negatively correlated with compl (r = −0.52, *p* < 0.05, Figure 2).

#### 3.1.3. Level of Spinal Lesion/Mobility

The majority of NB patients (35/58 (61%)) had lumbosacral spinal lesion, 15/58 (25%) had thoracolumbar and 8/58 (14%) sacral lesion level. We did not find statistically significant differences in NGAL and KIM-1 between the abovementioned groups. However, statistically significant differences were recorded in height-to-age z-score, serum and urinary Cr, eGFR Schwartz, and NGAL between the HS groups. More detailed data are shown in Table 3. Children from the 4HS group, described as community walkers, had the lowest urinary NGAL levels and uNGAL/Cr ratio in contrast to children with different stages of walking impairment from the remaining HS groups. In our studies, no significant difference was found among the HS groups for KIM-1 (Chi2 = 5.69, *p* = 0.13).

## 4. Discussion

To the best of our knowledge, this study is the first to focus on urinary biomarkers, KIM-1 and NGAL, as potential predictors of renal tubular injury in children with NB after MMC. Owing to adequate nephro-urological management in the last decade, the prognosis of MMC children has greatly improved, but the prompt recognition of renal damage in NB still remains a challenge. It is a largely asymptomatic process, and establishing the diagnosis currently depends on clinical tests, including elevated serum Cr and decreasing eGFR. Unfortunately, they are delayed and unreliable biomarkers of the future course of the disease for a variety of reasons. Cr depends on body muscle mass. In children with MMC, muscle wasting due to denervation is observed. What is more, MMC children often have impaired linear growth [14]. In these cases, Cr and precise estimation of eGFR may be impossible. There may be substantial inaccuracy when we compare eGFR between NB patients and the general population [1,15]. Recently, Cys has been shown to be a reliable marker in detecting renal deterioration in patients with NB [16,17]. It is not influenced by age, gender or muscle mass, consistent with our results. Additionally, it is not dependent on the patient’s level of physical activity assessed by HS. In our study, we do not reveal the differences in Cys and Cys-based eGFR between HS groups in contrast to Cr and eGFR Schwartz.

Our findings also reveal that the Schwartz equation gave a higher median eGFR compared to the Cys-based eGFR, likely due to the higher inaccuracy of Cr and height. These results are in good agreement with other studies [17] which have shown that lower eGFR in NB were observed when calculated using Cys than Cr, with the conclusion that Cys-based eGFR is a more sensitive parameter. However, serum Cr is still more popular, available and less expensive than Cyc in daily clinical practice. A clinical decision in the management of NB children made only with Cr and its associated eGFR may give the impression that overall kidney function is in the normal range, when in fact the opposite may be true. Moreover, they are primarily markers of glomerular disease and do not necessarily detect tubular dysfunction.

The presented study aimed to investigate early indicators of tubular damage in MMC patients. One of the inclusion criteria for our project was eGFR > 90 mL/min/1.73 m^2^ to choose NB patients without the biochemical signs of glomerular impairment assessed by common and widespread used renal parameters. Other objective methods of renal damage include image testing and urodynamic studies. Much research on risk factors of renal damage in patients with MMC has been conducted. The proposed risk factors included the identification of urinary tract abnormalities in, e.g., voiding cystourethrograms, ultrasonography, renal scintigraphy, or the presence of high-pressure bladder in urodynamics. However, the above-mentioned diagnostic tools can only identify upper tract abnormalities once they have already occurred. In our paper, the focus of our attention was on NGAL and KIM as markers that appear before the damage is present.

In our study, children with NB had significantly elevated uNGAL levels in comparison with healthy participants in contrast to uKIM-1. Additionally, girls have higher uNGAL levels compared to boys. This result is consistent with the observation by Pennemans et al. [18] who reported that in the group of healthy children, uNGAL levels were positively correlated with female gender in young children.

There are plenty of other factors that may influence the urinary levels of NGAL. Children with NB require clean intermittent catheterization which predisposes them to rUTIs. All patients from the study group were catheterized several times per day. In our study, higher uNGAL concentrations were observed in NB children with rUTI history. The most likely explanation of this result may be the greater expression of uNGAL by tubular epithelial cells in response to chronic inflammatory processes. There are several surveys conducted on the general pediatric population that show that uNGAL is higher in UTIs [19,20]. Only one study, performed by Foster et al. [21], focused on children with NB and indicated that uNGAL has a high predictive value for UTI in this population. However, it is not comparable to our research because the study group comprised NB children with active UTI or bacterial colonization of the urinary tract. In our study, the active UTI was an exclusion criterion. However, chronic inflammation may be the cause of fibroproliferative changes in the bladder wall including smooth muscle cell proliferation and hypertrophy. According to our results, uNGAL was increased in trabeculated, non-compliant bladder with a thickened wall. These factors may lead to high intravesical pressures during urine storage that impair its drainage into the bladder. High pressure requires more aggressive treatment to avoid further wall hypertrophy and potential deterioration of the bladder and upper urinary tract. The management must start before consequences of bladder dysfunction become apparent. On the basis of our results, we deduce that uNGAL may increase before the upper urinary tract damage is present or before the markers of glomerular function are able to detect it. uNGAL is released in response to tubular injury. Quite recently, considerable attention has been paid to the relation between tubulointerstitial damage and renal function decline. Several publications have documented that tubular dysfunction correlates better with renal deterioration than glomerular injury and may be a separate aspect of kidney pathology not fully reflected by glomerular biomarkers [22]. It is possible that in NB children with detected trabeculation and poor bladder compliance, tubular injury has occurred and is present but is not detectable because of the lack of tubular markers. This is the reason why uNGAL may become the potential tubular biomarker for predicting early renal dysfunction in NB.

An additional risk factor associated with rUTIs in the NB population is the presence of VUR. Several publications have appeared in recent years documenting the relationship between uNGAL and VUR but these results are not conclusive. Parmaksız [23] and Nickavar [24] indicate that uNGAL may be considered as a noninvasive diagnostic biomarker of primary VUR, especially of its long-term consequences such as renal scarring. However, our results are in good agreement with other studies which showed that presence of VUR did not change the uNGAL concentrations [25].

According to our results, the patient’s level of mobility is another factor affecting uNGAL. Wheelchair-dependent children had higher uNGAL levels than walkers. This finding suggests that children who are wheelchair-dependent have a higher risk of tubular dysfunction than others. Surprisingly, the parameters of glomerular function also differed but the median serum concentration of creatinine was the lowest and the creatinine-based eGFR was the highest in HS1 compared to other HS groups. The most likely explanation of this result is the fact that wheelchair-dependent patients have the most impaired linear growth and muscle atrophy. Especially in this group, the precise estimation of renal function with available parameters is impossible, so uNGAL may be the promising marker of renal function in this population of patients. On the basis of the promising findings presented in this paper, work on the remaining issues is still warranted to gain further insight on uNGAL and NB.

## 5. Conclusions

To conclude, we would like to highlight that:

1. Elevated levels of uNGAL could be considered a biomarker of tubular damage in children with NB due to MMC in contrast to uKIM-1.
2. Cystatin C is a more sensitive parameter of glomerular function in NB than creatinine.
3. Gender, history of recurrent UTIs, bladder trabeculation, wall thickening, and compliance may be factors influencing uNGAL concentration.

## Figures and Tables

**Table 1:** Demographic characteristics of patients with NB and control participants.

Variables	NB Patients *n* = 58	Control Participants *n* = 25	*p* Value
Gender: female/male *n* (%)	32 (55)/26 (45)	17 (68)/8 (32)	0.36
Median (minimum–maximum)			
Age (years)	9.2 (0.58–17.7)	12 (1–17)	0.07
Z-score: height-to-age	−0.9 (−5.1–3.1)	0.25 (−3.3–2.7)	0.01 *
Z-score: weight-to-age	−0.7 (−6–2.8)	−0.15 (−2.4–2.2)	0.08
Z-score: BMI (kg/m^2^)	−0.2 (−6–2.8)	−0.35 (−2.1–3)	0.95
Serum creatinine (mg/dL)	0.31 (0.18–0.88)	0.52 (0.2–0.85)	<0.001 *
Urinary creatinine (mg/dL)	52.1 (12.6–234.7)	129.5 (51–244)	<0.001 *
eGFR Schwartz (mL/min/1.73 m^2^)	157.5 (90–268)	117 (90–247)	<0.001 *

NB, neurogenic bladder; * *p* < 0.05.

**Table 2:** Biochemical parameters in patients with NB after MMC and control participants.

Variables	NB Patients *n* = 58	Control Group *n* = 25	*p* Value
Urinary KIM-1 (ng/mL)	0.46 (0–1.93)	0.62 (0–3.8)	0.1
Urinary KIM-1/Cr ratio (ng/mg)	0.76 (0–3.01)	0.54 (0–1.46)	0.08
Urinary NGAL (ng/mL)	10.3 (0.55–73)	1.32 (0–62)	<0.001 *
Urinary NGAL/Cr ratio (ng/mg)	27.1 (1.08–240.1)	0.98 (0–93)	<0.001 *

* *p* < 0.05.

**Table 3:** Comparison between HS groups.

Variables	HS1 Wheelchair Dependent	HS2 Walking Only in Therapy	HS3 Household Walkers	HS4 Community Walkers	Chi 2	*p* Value
*n* (%)	29 (50)	9 (16)	7 (12)	13 (22)		
z-score: height-to-age	−1.8(−5.1–3.1)	−1 (−4.3–1.4)	0.1 (−2–1.5)	−0.7 (−2.5–2)	9.48	0.02 *
Serum creatinine (mg/dL)	0.27 (0.18–0.73)	0.35 (0.2–0.64)	0.33 (0.24–0.88)	0.4 (0.23–0.76)	11.28	0.01 *
Urinary creatinine (mg/dL)	42 (12.6–114.1)	61.5 (24.3–234.7)	65.3 (32.6–75.81)	58.1 (19–182.1)	7.93	0.04 *
eGFR Schwartz (mL/min/1.73 m^2^)	178.5 (91.4–268.4)	167.6 (100–239.5)	146.55 (90–178.97)	123 (92.4–175.6)	9.28	0.02 *
Cystatin C (mg/L)	0.66 (0.18–0.83)	0.69 (0.54-0.85)	0.63 (0.59–0.83)	0.61 (0.4–0.75)	2.79	0.42
eGFR with cystatinC (mL/min/1.73 m^2^)	104 (84–278)	99 (90–123)	108 (90–116)	112 (91–162)	2.95	0.39
Urinary NGAL (ng/mL)	27.6 (1.28–73)	12.14 (4.87–51.75)	31.6 (1.87–51.14)	4.68 (0.55–28.82)	11.17	0.01 *
Urinary NGAL/Cr ratio (ng/mg)	37.14 (4.57–240.1)	26.61 (3.19–140.6)	58.03 (3.37–96.9)	9.2 (1.08–45.7)	10.63	0.01 *

* *p* < 0.05.

## Data Availability

The data presented in this study are available on request from the corresponding author. The data are not publicly available for ethical and privacy reasons.
